# Supplementary material for: Deletion of wfs1 Impairs Oligodendrocyte Precursor Cells Dorsal Distribution and Myelination Through the wfs1-hmgcs1 Axis in Zebrafish
Source: Biology (Basel). 2026 Aug 16;15(16):1402. doi: 10.3390/biology15161402 (PMC13510151; doi:10.3390/biology15161402)
Supplement: Supplementary file 1 [file biology-15-01402-s001.zip › File S1-Transcriptome sequencing data and differentially expressed genes (DEGs) in wfs1-- zebrafish at 5 dpf/2.KEGG/wfs1KOvsWT/GSEA/wfs1KO/FATTY_ACID_BIOSYNTHESIS/FATTY_ACID_BIOSYNTHESIS(DRE00061).html]

Details for gene set FATTY\_ACID\_BIOSYNTHESIS(DRE00061)[GSEA]

|  || Dataset | deg.sample |
| Phenotype | sample.cls |
| Upregulated in class | wfs1KO |
| GeneSet | FATTY\_ACID\_BIOSYNTHESIS(DRE00061) |
| Enrichment Score (ES) | 0.34425613 |
| Normalized Enrichment Score (NES) | 1.3049469 |
| Nominal p-value | 0.032719836 |
| FDR q-value | 0.28553855 |
| FWER p-Value | 0.957 |
Table: GSEA Results Summary

  

Fig 1: Enrichment plot: FATTY\_ACID\_BIOSYNTHESIS(DRE00061)      
 Profile of the Running ES Score & Positions of GeneSet Members on the Rank Ordered List

  

| PROBE | DESCRIPTION (from dataset) | GENE SYMBOL | GENE\_TITLE | RANK IN GENE LIST | RANK METRIC SCORE | RUNNING ES | CORE ENRICHMENT || 1 | 559403\_1 | acaca |  |  | 1982 | 1.374 | 0.0584 | Yes |
| 2 | 103909985 | LOC103909985 |  |  | 3098 | 1.013 | 0.1123 | Yes |
| 3 | 393622 | acsl4a |  |  | 3304 | 0.972 | 0.1912 | Yes |
| 4 | 562292\_2 | acsf3 |  |  | 4884 | 0.696 | 0.2027 | Yes |
| 5 | 100006023 | acsbg1 |  |  | 5003 | 0.680 | 0.2588 | Yes |
| 6 | 559403 | acaca |  |  | 5108 | 0.666 | 0.3140 | Yes |
| 7 | 447860 | acsl5 |  |  | 5835 | 0.604 | 0.3443 | Yes |
| 8 | 402954 | cbr4 |  |  | 9038 | 0.365 | 0.2758 | No |
| 9 | 562292\_1 | acsf3 |  |  | 9095 | 0.360 | 0.3057 | No |
| 10 | 550550 | mecr |  |  | 9422 | 0.339 | 0.3252 | No |
| 11 | 559656 | acsl3b |  |  | 11481 | 0.218 | 0.2797 | No |
| 12 | 566863 | acsl6 |  |  | 12701 | 0.154 | 0.2550 | No |
| 13 | 568096 | olah |  |  | 13379 | 0.122 | 0.2444 | No |
| 14 | 559001 | fasn |  |  | 15355 | 0.030 | 0.1850 | No |
| 15 | 334561 | acsl4b |  |  | 16648 | -0.022 | 0.1464 | No |
| 16 | 445175 | acsl1b |  |  | 16699 | -0.023 | 0.1468 | No |
| 17 | 564649 | osxm |  |  | 17307 | -0.045 | 0.1317 | No |
| 18 | 555308 | acsl1a |  |  | 18027 | -0.077 | 0.1160 | No |
| 19 | 556236 | acacb |  |  | 18028 | -0.077 | 0.1227 | No |
| 20 | 564111 | mcat |  |  | 18915 | -0.118 | 0.1053 | No |
| 21 | 567157 | acsl3a |  |  | 19451 | -0.142 | 0.1010 | No |
| 22 | 335159 | acsbg2 |  |  | 19541 | -0.145 | 0.1109 | No |
| 23 | 100332846 | acsl2 |  |  | 21878 | -0.261 | 0.0605 | No |
| 24 | 101884755 | LOC101884755 |  |  | 22823 | -0.315 | 0.0585 | No |
| 25 | 555308\_1 | acsl1a |  |  | 24418 | -0.416 | 0.0450 | No |
| 26 | 64815 | hsd17b8 |  |  | 25500 | -0.485 | 0.0536 | No |
| 27 | 562292 | acsf3 |  |  | 27857 | -0.723 | 0.0431 | No |
| 28 | 559001\_1 | fasn |  |  | 29124 | -0.950 | 0.0868 | No |
Table: GSEA details [plain text format]

  

Fig 2: FATTY\_ACID\_BIOSYNTHESIS(DRE00061)      
 Blue-Pink O' Gram in the Space of the Analyzed GeneSet

  

Fig 3: FATTY\_ACID\_BIOSYNTHESIS(DRE00061): Random ES distribution      
 Gene set null distribution of ES for **FATTY\_ACID\_BIOSYNTHESIS(DRE00061)**
